# Treatment with medication for patients with psychosis within 2 days during waiting in the accident and emergency department and its correlation with length of in-patient stay: retrospective database study

**DOI:** 10.1192/bjo.2024.804

**Published:** 2024-11-11

**Authors:** Hannah Pasha Memon, Nacharin Phiphopthatsanee, Elliot Hampsey

**Affiliations:** South London and Maudsley NHS Foundation Trust, London, UK; Institute of Psychiatry, Psychology, & Neuroscience, King's College London, London, UK; Faculty of Medicine, King Mongkut's Institute of Technology Ladkrabang, Bangkok, Thailand

**Keywords:** Antipsychotics, in-patient treatment, clinical outcomes measures, psychotic disorders/schizophrenia, liaison psychiatry

## Abstract

**Background:**

One of the ‘critical goals’ for psychiatric liaison services is reducing hospitalisation. Psychotropic medication is a treatment for psychosis, although research determining the efficacy of early medication administration is lacking.

**Aims:**

To determine whether commencing psychotropic medication within 2 days of psychiatric liaison input in the accident and emergency (A&E) department is correlated with length of in-patient psychiatric admissions for patients with psychosis.

**Method:**

We gathered data on patients presenting to A&E sites covered by South London and Maudsley (SLaM) National Health Service Trust, who were subsequently admitted to and discharged from SLaM psychiatric in-patient wards with discharge diagnosis of psychosis between 2015 and 2020. The analysis set comprised 228 patients waiting in the A&E department under psychiatric liaison care for ≥2 days, of which 140 were started on medication within those 2 days (group A) and 88 were not (group B). Group A was divided into A1 (patients restarted on previous psychotropic medication taken within 1 week) and A2 (others, including those new to psychotropic medication or with past usage).

**Results:**

Although Kaplan–Meier survival curves with log-rank tests demonstrated no statistically significant difference of in-patient admission duration between groups A and B or groups B1 and B2, further analysis revealed that subgroup A1 had statistically significant shorter admissions than group B (*P* = 0.05).

**Conclusions:**

Restarting patients with psychosis on medication they were taking within the week before A&E department attendance, within 2 days of arrival at the A&E department, is associated with statistically significant shorter admissions. The limitation is a relatively small sample size.

Following a report released by the Care Quality Commission in 2019 regarding mental health patients in the accident and emergency (A&E) department waiting for 12 h for an in-patient psychiatric bed,^1^ the National Health Service (NHS) England began working towards better access to mental health services for all,^2^ with liaison psychiatric services finding themselves in the spotlight. In 2020, the Department of Health and the NHS began working towards creating plans to fund services that ensure the waiting time for mental health patients trying to access mental health help is shortened.^3^ As of 2021, there has been a significant increase in the availability of 24/7 psychiatric liaison services (80% of general hospitals, compared to 39% in 2016)^4^ and a growing proportion of mental health attendances were admitted to psychiatric units out-of-hours.^5^

However, despite these efforts, the number of available NHS in-patient psychiatric beds does not always meet requirements,^6^ causing some psychiatric patients to wait in the A&E department for prolonged periods until a psychiatric in-patient bed becomes available. Our study focuses on this cohort of patients who appear to be in management limbo: not safe enough to be discharged home, but also not in an appropriate setting for holistic psychiatric management.

## Current guidance for liaison services

One of the integral roles of liaison psychiatric services is to provide psychiatric assessment and treatment within the A&E department and to reduce both admission pressures and disease burden.^7^ National Institute for Health and Care Excellence (NICE) guidance for liaison services released in 2017 states that psychiatric illnesses should be diagnosed and treated ‘early’ to achieve the critical goal of reducing hospital stay for psychiatric patients.^8^ The Royal College of Psychiatrists’ guidelines for liaison services indicate that liaison services should respond to emergency referrals within 60 min, urgent referrals within 24 h and non-urgent mental health referrals within 2 days.^9^ Treatment can take various forms, from assessment and management of self-harm to provision of specialised services.^10^

Although the use of medication to manage symptoms is one of the most important tools in managing psychiatric patients in the A&E department,^11^ clear timeframes regarding the commencement of psychotropic treatment for psychiatric patients presenting to liaison settings are currently lacking. In addition, with the NHS Mental Health Implementation Plan^12^ aiming to reduce in-patient psychiatric admission to less than 32 days, there is little in the way of guidance on how to achieve this.

## Psychosis

There appears to be a consensus on the umbrella term ‘psychosis’, but some debate exists around the illnesses that are categorised as psychotic illnesses. In the book *Pharmacological Treatment of Mental Disorders in Primary Health Care* by the World Health Organization (WHO), psychosis is defined as delusions, hallucinations, bizarre behaviour and loss of contact with reality; it mentions schizophrenia as the most common primary psychotic disorder; bipolar disorder is separately defined as an illness with episodes of mania and depression.^13^ ICD-11 by WHO does not provide a definition of psychosis; it mentions schizophrenia as a primary psychotic illness, characterised by significant impairments in reality testing and alterations in behaviour, with positive symptoms such as delusions, hallucinations, disorganised thinking and other psychomotor disturbances.^14^ It allows for a diagnosis of mania with psychotic symptoms and mania without psychotic symptoms, and the same for depressive episodes and bipolar affective disorder episodes. The *Oxford Handbook of Psychiatry* mentions that psychotic illnesses encompass schizophrenia and its related psychotic illnesses, as well as mood disorders with psychotic features.^15^

Notwithstanding the variable categorisation of psychotic illnesses in textbooks, the literature consistently demonstrates that a diagnosis of a psychotic illness or bipolar affective disorder is associated with prolonged in-patient stays.^16^

Psychotropic drugs or psychoactive drugs are medications that affect mental processes, for example, perception, consciousness, cognition or mood and emotions.^17^ These, along with psychosocial interventions, are cornerstones of the management of schizophrenia and other psychotic disorders,^18^ with some evidence supporting an immediate commencement of psychotropic medication for an acute psychotic episode.^13,19^

Apart from patient factors, it has been shown that service-level factors such as prolonged wait time can have a negative impact on patient experience and safety, including potential deterioration in patients’ conditions.^20^ However, there is a lack of research evidence on how psychiatric admissions can be shortened and there are no guidelines on timeframes for when to start psychotropic medication for favourable patient outcomes.

## Aim of the study and hypothesis

This study aims to determine whether starting medication in the A&E department within 2 days is correlated with a shorter length of in-patient stay for patients with psychosis who awaited psychiatric admission in the A&E department for 2 days or longer. In other words, our study determines whether there is a relationship between early medication administration for patients with psychosis and shorter psychiatric admissions. We determined that 2 days would be an appropriate timeframe for the purpose of our study, as each patient, including those who presented over a weekend, would have been reviewed by a senior psychiatrist within 2 days. We hypothesised that patients who had psychotropic medication started under liaison care within 2 days of A&E department wait time would have shorter in-patient stays and be discharged faster than their counterparts.

## Method

### Study design

#### Geographical area and time period

South London and Maudsley (SLaM) NHS Foundation Trust provides mental health services across south-east London, covering the four boroughs of Croydon, Lambeth, Lewisham and Southwark, with an estimated population (2015) of over 1.3 million.^21^ There are four A&E sites covered by SLaM NHS Trust where patients attend for emergency mental healthcare: Croydon University Hospital A&E, King's College Hospital A&E, St Thomas’ Hospital A&E and University Hospital Lewisham A&E. Data were gathered using the Clinical Record Interactive Search (CRIS) system, which is an anonymised research database containing clinical record data from across SLaM. We gathered data for patients attending all four SLaM A&E sites from 1 January 2015 to 31 December 2020.

#### Diagnoses inclusion criteria

Psychoses diagnoses included for the study were schizophrenia, schizophrenia-related disorders, delusional disorders, non-organic psychoses, affective disorders with psychotic symptoms and manic episodes without psychotic symptoms. A full list of included ICD-11 codes can be found in Appendix A.

#### Other inclusion criteria

Data were gathered on patients that attended a SLAM A&E site between 1 January 2015 and 31 December 2020 who met the following criteria:

- required psychiatric admission and waited in A&E longer than 48 h or 2 days for an in-patient psychiatric bed (start of clock: first liaison note entry and/or liaison team acceptance form; close of clock: 2 days or 48 h from first acceptance or first liaison clinical note entry, evidenced by the continuation of reviews notes by liaison personnel); and
- were subsequently admitted to one of the SLaM psychiatric in-patient wards following this attendance; and
- were discharged from that psychiatric ward with a final discharge diagnosis of a psychosis presentation.

#### Exclusion criteria

Excluded from the study were the following:

- patients with active alcohol use requiring alcohol detoxification;
- patients with reported drug use at the time of presentation to the A&E department;
- patients with organic psychosis by other causes, for example, delirium secondary to a medical illness;
- patients for whom complete data were not available, for example, patients admitted to a hospital bed outside SLaM NHS Trust (NHS or private) or patients admitted directly to a SLaM NHS Trust bed without first attending an A&E liaison site covered by SLAM NHS Trust;
- patients admitted to place-of-safety units;
- patients with concurrent physical conditions requiring medical or surgical interventions, since such patients would, as per A&E department protocols, require a general hospital medical or surgical bed initially (as opposed to a psychiatric bed).

#### Information available

Patient information accessible for our research included the following:

- demographic data (dates of birth, gender and ethnicity);
- any clinical notes on the SLaM NHS Trust electronic patient record system before A&E liaison clinical entries, for example, notes written by community staff nurses or care-coordinators arranging for patient to attend A&E or discussing the need for psychiatric admission;
- A&E department liaison referrals, liaison acceptance and discharge dates with timestamps, any clinical notes during and after the A&E department stay, Mental Health Act status, security status, observation status and results of capacity assessments;
- dates and times referred to and accepted for in-patient psychiatric admission, with all subsequent in-patient notes and discharge notes from the ward with dates, timestamps and discharge diagnoses.

#### Medications

To study the effect of early medication commencement, we gathered information on all medications prescribed as part of psychiatric management in A&E. All medications that have a psychotropic impact (e.g. antidepressants, mood stabilisers, antipsychotics, benzodiazepines, pregabalin) were included if prescribed for the purpose of having a psychotropic effect. Psychotropic medications prescribed for non-psychiatric symptoms (e.g. amitriptyline for neuropathic pain) were not considered for inclusion in the study. In our study, we encountered no patients started on any psychotropic medication for any other reasons than for its psychotropic effect.

#### Ethical approval

The CRIS system was approved as a data-set for secondary analysis by the Oxford South Central C Research Ethics Committee (REC; REC reference: 23/SC/0257). This ethical approval means that individual research projects are not required to apply for individual project ethics. Instead, applications to use CRIS are reviewed and approved by the patient-led CRIS Oversight Committee.

### Data management

The analysis set consisted of 228 patients who waited in the A&E department for above 2 days whilst fulfilling the above criteria, of which 140 patients were started on psychotropic medication within 2 days of liaison psychiatry input in the A&E department (group A), and 88 were not (group B). The groups were further sub-divided into subgroups A1 (patients who were restarted on any of their psychotropic medication in the A&E department that they had stopped within the past week) and A2 (all other patients, including psychotropic-naïve patients as well as patients who had had psychotropic medication in the near or distant past), and subgroups B1 (patients not started on psychotropic medication in the A&E department, who were taking psychotropic medication within the week before A&E department attendance) and B2 (all other patients, including psychotropic-naïve patients as well as patients who had had psychotropic medication in the near or distant past).

**Figure 1:**
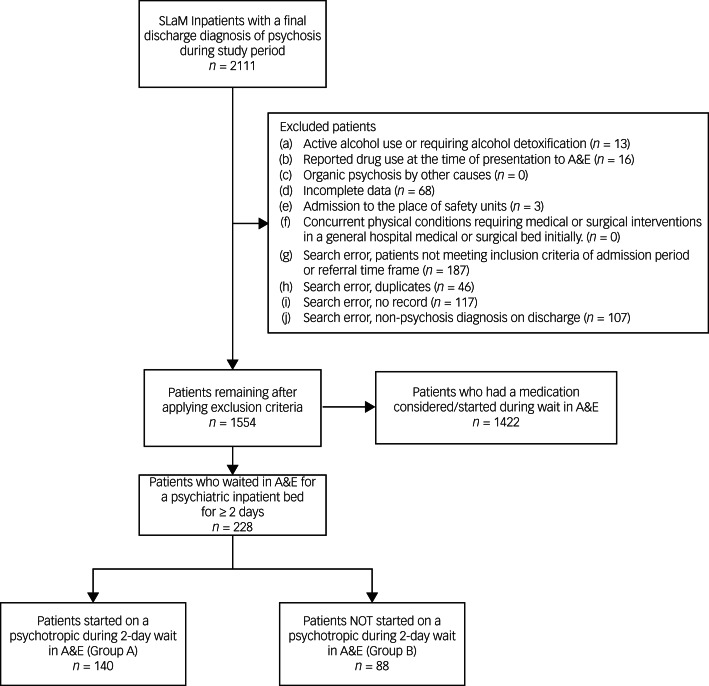
Flow diagram overviewing patient record collection, exclusion and grouping. SLaM, South London and Maudsley [National Health Service Trust]; A&E, accident and emergency [department].

**Figure 2:**
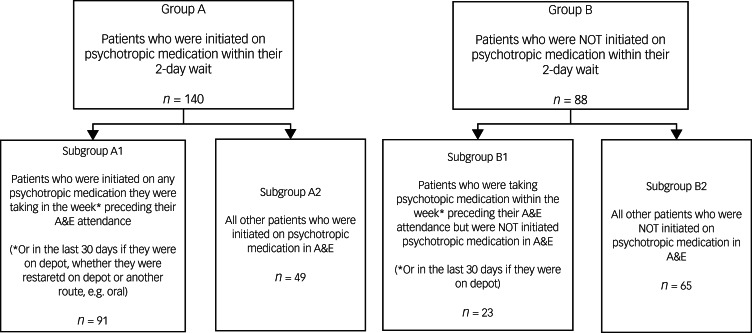
Flow diagram overviewing subgrouping of patients. A&E, accident and emergency.

When a patient on a long-acting injectable antipsychotic (depot) presents to the A&E department with psychiatric symptoms, he/she is clearly unwell and in need of further psychiatric management, of which pharmacological treatment is considered one of the effective options. For patients on depots, the doses were expected to have been administered within 30 days before A&E department attendance. No patients in our study were on 3-monthly long-acting injections. Patients administered the same medication, regardless of the formulation, would be assigned to A1. For example, a patient on aripiprazole depot who was started on oral or intramuscular aripiprazole during the A&E department wait would fall into subgroup A1. Patients on paliperidone depot who were given oral risperidone in the A&E department would also be assigned to subgroup A1. Patients who were not started on any medication in the A&E department (group B) but had a depot dose within the last 30 days, were assigned to B1. If there was no evidence that a depot dose was administered within 30 days before A&E department attendance, the patients would then be assigned to group B2. Details on the clinical records data obtained from CRIS are overviewed in Fig. 1, with subgrouping detailed in Fig. 2.

### Statistical analysis

All statistical analyses were performed using IBM SPSS Statistics (version 27 for Windows). Demographic, epidemiological and treatment information is described below using summary statistics. Chi-squared tests determined if there were any statistically significant (*P* < 0.05) differences among any of the demographic, epidemiological and treatment information data, both between groups and for the entire cohort.

Kaplan–Meier estimator^22^ and Mantel–Cox log-rank tests^23^ were used to model the cumulative probability of discharge from hospital between groups A and B, subgroups A1 and B and subgroups A2 and B. The log-rank test also compared survival distributions of the length of in-patient stay between the demographic variables collected. These tests were selected as the primary outcome concerns time to an event, that is, discharge. Furthermore, our data satisfies the necessary assumptions for these tests, namely that (a) any potential censoring is unrelated to prognosis (in this case there is no censoring as all patients achieved discharge), (b) survival probabilities are equal for those entering the patient pool at different times and (c) that the times events were recorded as happening were accurate. A Cox regression^24^ was also employed to determine if any observed differences between the two groups were mediated by any demographic variables (Table 1) or features of in-patient stay (Table 2).

**Table 1:** Demographic information for the full data-set (*n* = 228)

Demographic factor	Group A (*n* = 140)	Group B (*n* = 88)
Age	38.6	44.2
Male/female	60.7/39.3%	62.5/37.5%

NB. Ethnic background information listed in Appendix C owing to heterogeneity.

**Table 2:** Frequencies of factors related to a patient's in-patient stay. Rates of detention under the Mental Health Act were high across groups (70.8–87.8%), with the need for either 2:1 or 1:1 monitoring also very high. There were no statistically significant differences between groups A and B or subgroups with regards to in-patient stay factors (*P* ≥ 0.1)

	Group		Subgroup			
In-patient Stay Factors	A (*n* = 140)	B (*n* = 88)	A1 (*n* = 91)	A2 (*n* = 49)	B1 (*n* = 23)	B2 (*n* = 65)
Detained (MHA)	77.1% (108)	80.7% (71)	71.4% (65)	87.8% (43)	73.9% (17)	83.1% (54)
Required observation	92.9% (130)	89.8% (79)	92.3% (84)	93.9% (46)	91.3% (21)	89.2% (58)
Medication started	Appendix B	N/A	Appendix B	Appendix B	N/A	N/A
Length of stay (days)	Range 1–341 Mean 46.6 Median 26	Range 2–405 Mean 54.4 Median 35.5	Range 1–264 Mean 40.2 Median 26	Range 1–341 Mean 59.1 Median 36	Range 2–331 Mean 50.6 Median 25.5	Range 2–405 Mean 55.8 Median 37

MHA, Mental Health Act.

Because of the small number of comparisons (four in total), all of which were planned from *a priori* hypotheses about our groups, tests to control for multiple comparisons were not conducted.

## Results

A total of 228 patients were included in the analysis, with 140 allocated to group A and 88 to group B. These groups were further divided into subgroups: group A into A1 with 91 patients and A2 with 49 patients, and group B into B1 with 23 patients and B2 with 65 patients. The demographic information of the sample is described in Table 1, with full details of the ethnic makeup of the sample presented in Appendix C. Chi-squared tests confirmed the homogeneity of the sample, with no demographic factor significantly more or less prevalent in either group (all *P* ≥ 0.05).

**Figure 3:**
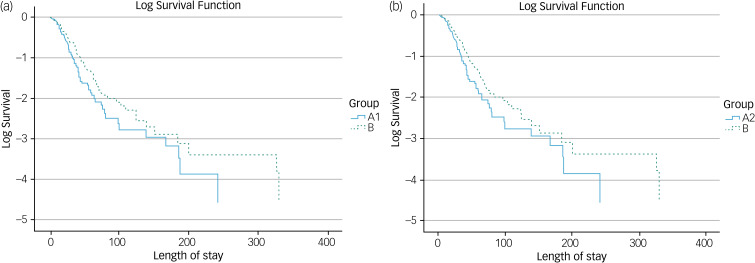
(a) Kaplan–Meier survival plot for time to discharge between subgroup A1 and group B. (b) Kaplan–Meier survival plot for time to discharge between subgroup A2 and group B.

Figure 3(a) shows the Kaplan–Meier survival plot for time to discharge between group A1 patients and group B patients. The Mantel–Cox log-rank test returned a significant difference in length of stay (*P* = 0.05) between the groups, with a chi-square value of 3.845 (d.f. = 1). Figure 3(b) shows the Kaplan–Meier survival plot for time to discharge between group A2 patients and group B patients. The Mantel–Cox log-rank test returned a non-significant difference in time to discharge (*P* = 0.241) between the groups, with a chi-square value of 1.373 (d.f. = 1).

Although the length of stay for group A patients (those who were started on medication within 2 days) was lower than that for group B (patients not started on any medication in the A&E department), this difference was not statistically significant at the 0.05 level. However, group A1 patients, that is, patients who were restarted on the psychotropic medication that they were taking within the week before A&E attendance, within 2 days of A&E department wait times, had a statistically significant shorter duration of in-patient stay. Medication restarted may be an antipsychotic, a mood stabiliser, a benzodiazepine or an antidepressant, treatment that they had not adhered to, within the week before attending the A&E department. This reiterates the findings from other literature, which highlight the impact of medication non-adherence on relapse and rates of readmission to hospital.^25,26^ Within group B, while the average length of in-patient stay was shorter in subgroup B1 (patients who were taking psychotropic medication within the week preceding their A&E department attendance but were not initiated psychotropic medication in the A&E department) than in subgroup B2 (all other patients who were not initiated on psychotropic medication in the A&E department), this difference did not reach statistical significance.

A Cox regression model was employed to assess the explanatory value of covariates on length of stay. The results of the analysis indicate that being restarted on a medication the patient was taking within the week before A&E attendance, within 2 days of arriving in A&E, being associated with a shorter length of stay was particularly true for male patients (chi-square = 8.489, d.f. = 2, *P* < 0.014).

It may be of interest to readers that we also collected data on commencement of medication practices for all patients within the study period who attended the A&E department. These patients fall into the ‘patients who had medication considered in A&E’ category (see Fig. 1). This group of patients were assessed and waited in the A&E department for any given time period, from a few hours to over 2 days; they either had medication started, or discussions were had around starting medication. This highlights that considering medication and/or starting medication in the A&E department is a common tool utilised in liaison settings, but there is no homogeneity or clarity of rationale regarding who has medication started and when.

## Discussion

### Waiting in the A&E department for over 2 days

The group of patients assessed in this study were waiting in the A&E department for over 2 days for an in-patient psychiatric bed, with acute psychotic symptoms. It is difficult to comment on the qualitative experience these patients had of the prolonged stay in the A&E department. Although these patients had a multidisciplinary team who tried to optimise their care to the best level possible in that setting, A&E departments are well-known to be chaotic, stress-provoking and frightening. The added goal of this study, therefore, is to ensure that this cohort of patients receive care that is as close to the standard of care on in-patient psychiatric wards as possible, and that future studies focus on the potential negative psychological and psychiatric impact of waiting in the A&E department, if any.

### Group A versus B

**Figure 4:**
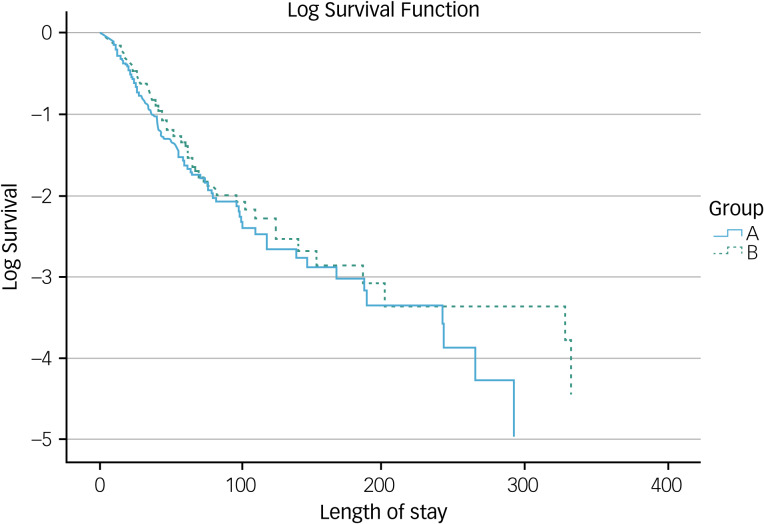
Kaplan–Meier survival plot for time to discharge between groups A and B. The data is entirely uncensored as all data points reached the ‘event’, i.e. discharge, with the latest discharge in the sample occurring after 405 days. The Kaplan–Meier survival curves with Mantel–Cox log-rank test determined that although group A had a shorter in-patient stay than group B, this was not statistically significant (*P* = 0.214, χ² = 1.373).

As shown in Fig. 4, the length of stay does appear to be consistently slightly worse for group B than group A, but ultimately this difference was not statistically significant. We decided to include participants with any length of stay in our data-set, opting against ‘cleaning’ any very long length of stay data from our data-set so as to give an accurate representation of the admission durations of this patient cohort. It is worth considering that the medications someone is taking are unlikely to remain relevant as other issues cause delays in discharge, for example, putting appropriate follow-up care in place or finding an appropriate discharge destination. It could be argued that a more realistic timeframe for evaluating the impact of medication would be 2–4 weeks from initiation, by which most psychotropic medication would have taken effect. Future research could consider reanalysing this question with special focus given to the 2–4 week period after initial medication administration to obtain a clearer picture of the impact of the medication titration itself.

### Subgroup A2

While commencing patients with psychosis on psychotropic medication they were previously on, within 2 days, has some effect on reducing the duration of in-patient admission, the effect did not reach statistical significance for patients who were not taking the medication within the past 1 week when they attended the A&E department (subgroup A2). There are several plausible explanations for the observed limited effect in this subgroup.

First, not recently taking psychotropic medication can reflect a wide gamut of psychiatric history ranging from acutely developed (which is why the patient is psychotropic-naïve) to treatment-resistant psychosis. The lack of homogeneity in this group in terms of the patients’ history of psychiatric medication use may be one reason why this group did not reach statistical significance.

For psychotropic-naïve patients, the trials and errors to achieve an appropriate treatment regimen and the time required for the gradual dose titration of psychotropic medication, such as an antipsychotic, antidepressant or mood stabiliser, are likely longer. This period tends to be shortened if the clinician is guided by the patient's previous response to medications or if the patient has previously partially adhered to treatment with a medication, as in subgroup A1.

Second, patients with psychosis in this subgroup included those who were newly commenced on just a sedative such as clonazepam or promethazine during their 2-day wait. It is worth considering that benzodiazepine use theoretically would be unlikely to alter the trajectory of a psychotic illness; however, subgroup A1 also included patients who were restarted on just their benzodiazepine during their 2-day wait (see Appendix B).

In conclusion, it is worth noting that despite the aforementioned inadequacies of this subgroup, the probability of discharge was consistently higher for those who received medication within 2 days of arriving within the A&E department compared to those who did not.

### Subgroups B1 and B2

Although the average duration of in-patient stays was shorter in subgroup B1 compared to that of B2, this difference was not statistically significant. This might be explained by the relatively shorter duration of untreated psychosis before admittance to hospital. A longer duration of untreated psychosis is found to be associated with poorer outcomes in schizophrenia, in both the short and long term across a number of domains: symptoms severity, remission rates, risk of relapse, global functioning and quality of life^27^; hence, it is worth mentioning that the patient subgroup whose outcomes could be most positively affected by the results of our study would be subgroup B1, that is, patients who were taking psychotropic medication up to 1 week before attending the A&E department. Restarting these patients on their medication during their 2 days’ wait would have them assigned to subgroup A1, leading to shorter in-patient stays.

### Prescribing preferences

It is noteworthy that the most commonly prescribed (newly started or restarted) antipsychotic was olanzapine, and the most commonly prescribed benzodiazepine for both groups (A1 and A2) was clonazepam, indicating clinicians’ preference for these two psychotropics over others in the A&E department. One of the reasons for this might be the availability of such medications in A&E department settings. In the UK, olanzapine is also available as an intramuscular injectable, which might account for its preferential use in the A&E department, where patients might be acutely unwell and refuse oral medication. According to a large meta-analysis examining the efficacy and tolerability of antipsychotics in schizophrenia, olanzapine was amongst the most highly effective antipsychotics, associated with a reduction in positive psychotic symptoms, negative psychotic symptoms and depressive symptoms (others being clozapine, amisulpride and, to some extent, sulpiride and risperidone, all of which are less widely available in the A&E department).^28^ It is also less associated with acute side effects, such as Parkinsonism, when compared to other widely available injectable antipsychotics such as haloperidol. Olanzapine's sedative side effects might also be desirable in the A&E department, where there are limited resources to manage agitated patients with psychosis. Although there is no evidence supporting the superiority of clonazepam to other benzodiazepines in psychosis, its availability and long half-life might account for its preferential use in A&E department settings.

### Barriers to prescribing practices

As this study concludes that timely recommencement of pre-existing prescriptions to shorten in-patient stay is essential, it is pertinent to reflect on the reasons why medication is not commenced as soon as patients attend the A&E department, particularly when they had been taking that medication before attendance. Is there a lack of access to pharmacy services and are previous prescriptions not identified quickly, that is, are more specialist on-call pharmacists needed? Are there problems with obtaining prescription information across the mental health trust/physical trust/primary care computer interface? In the case of psychotropic-naïve patients, is there is a lack of confidence by liaison doctors to prescribe? How common is it for liaison doctors to decide that a period of assessment without medication is the safest course of action and is that justifiable? These are some of the questions that need answers in future studies. In the case of the patient cohort in our study, even where patients’ psychotropic medication information was available, it was observed that the medication was often not commenced, with no clear rationale documented for non-continuation.

### Use of Mental Health Act and enhanced observations

There was no significant difference in the proportion of patients requiring detention under the Mental Health Act 1983 or enhanced observations (referring to the constant presence of security personnel or at least one healthcare professional by a patient's bedside to ensure the safety of the patient or the public, or to reduce the absconsion risk). These parameters reflect the comparable level of agitation and insight into illness (which has an implication on patients’ capacity to consent to voluntary admission and treatment) between each group. There was also no significant difference between groups with regard to ethnicity or gender.

### Demographics

Evidence consistently suggests that Black ethnic groups are more likely to be admitted to hospital under the Mental Health Act,^29^ which is associated with longer duration of in-patient stays.^30^ However, in our sample, there was no significant difference between groups with regard to ethnicity, although the high heterogeneity of ethnic background categories recorded on the CRIS database prevented meaningful analysis in this study (see Appendix C). There was also no difference between the groups in terms of patient gender, meaning demographic factors are unlikely to have confounded the results. Other demographic factors, such as income, education and marital status, which could have potentially confounded the results, were not consistently available in the clinical records; therefore, the analysis of these factors was not possible.

It was noteworthy that the Cox regression returned that reduced length of stay after receiving medication during the patient's 2-day wait in the A&E department was particularly evident for male patients in subgroup A1. It is unclear as to why this was the case; previous literature has shown considerable differences between the genders in terms of the presentation of schizophrenia as well as response to treatment, with women faring better than men.^31^ We therefore interpreted this finding as incidental.

### Limitations

The primary limitation of our study is the relatively small sample size from a specific part of London; hence, immediate generalisations cannot be made to patients with psychosis in other populations, especially those in less urbanised areas, as London is a very metropolitan area. Future research reproducing our results in a larger number of patients across urban and less urbanised zones would be beneficial.

The small sample size also limits our ability to perform subgroup analyses between different diagnoses, indications for medication, medications themselves or their formulations. Moreover, information regarding potential confounding variables other than ethnicity and gender was unavailable, limiting our ability to consider these factors.

It may be argued that psychotropic-naïve patients with psychosis are unlikely to be prescribed psychotropic medications in the A&E department, or that not prescribing medication in such a setting would allow for further assessment or maybe for patient safety. It is worthwhile to remind ourselves that a decision to prescribe a psychotropic agent does not render psychological and/or psychosocial interventions unnecessary,^32^ and while individual clinical situations are the best guides for clinicians making decisions on whether to withhold or to prescribe medication, the necessity of psychiatric admission reflects the severity of the psychotic presentation. Our study does not attempt to encourage psychiatrists to prescribe medication where it is clinically not appropriate; rather, it indicates where it may be clinically appropriate to do so. For future research, it would be useful to study the impact of antipsychotics alone on patients with psychosis who waited in the A&E department.

For the purpose of our study, it was necessary to base the analysis on the assumption that treatment documented as prescribed in SLaM NHS Trust liaison notes was administered by A&E department staff, although this may not have always been the case. Whilst availability and completeness of data appeared to be reliable, the potential for inconsistencies between documentation of medication prescriptions in SLaM NHS Trust liaison psychiatric notes versus acute Trust A&E department notes highlights the need for reproducing this study in a larger cohort to bolster the reliability of the results.

### Further implications

This is the first study that intends to analyse the effect of the administration of medication given in psychiatric liaison care in the A&E department on the duration of psychiatric admission. It is also the first study that attempts to offer a timeframe by which medication should be started for a positive clinical and treatment outcome for patients with psychosis.

The results showed that being started on psychotropic medication in the A&E department within 2 days was not itself associated with a shorter length of psychiatric admission. However, the results do outline a more nuanced finding, that is, restarting patients with psychosis on medication that they were established on within the past week of A&E department attendance leads to shorter hospital stays if started within 2 days of A&E department wait times. Medication restarted, be it a mood stabiliser, antipsychotic, antidepressant or benzodiazepine, helped in reducing in-patient admission duration. Subgroup A2, patients newly initiated on the same groups of medication, did not meet statistical significance.

These results make a strong case for recommencement of psychotropic medication within 2 days for patients with psychosis who present to the A&E department having stopped their medication within the past week. Such an intervention in emergency settings may have an implication on the allocation of limited resources, such as beds in psychiatric units, and will likely help reduce the duration of in-patient stays or may even divert patients from being admitted to hospital altogether.

## Data Availability

The data that support the findings of this study are available from the corresponding author, N.P., upon reasonable request.

## Appendix A

### Final discharge diagnoses included in the study with ICD-11 codes

1. Schizophrenia (F20)
2. Schizotypal disorder (F21)
3. Delusional disorders (F22)
4. Acute and transient psychotic disorder (F23)
5. Induced delusional disorder (F24)
6. Schizoaffective disorder and related episodes (F25)
7. Other non-organic psychotic disorder (F28)
8. Unspecified non-organic psychosis (F29),
9. Manic episode without psychotic symptoms (F30.1) and with psychotic symptoms (F30.2)
10. Bipolar affective disorder current episode manic without psychotic symptoms (F31.1) and with psychotic symptoms (F31.2)
11. Bipolar affective disorder current episode severe depression with psychotic symptoms (F31.5)
12. Severe depressive episode with psychotic symptoms (F32.3)
13. Recurrent depressive disorder current episode severe with psychotic symptoms (F33.3)

## Appendix B

### Medications initiated in groups A1 and A2

**Table:**

Patients who had medication started in the A&E department	
Group A1	Group A2
**Antipsychotics (88)** Amisulpride (5) Aripiprazole (14) Clozapine (9) Haloperidol (1) Flupenthixol (1) Lurasidone (3) Olanzapine (39) Paliperidone depot (1) Quetiapine (6) Risperidone (8) Sulpride (1)	**Antipsychotics (40)** Amisulpride (1) Aripiprazole (4) Clozapine (1) Haloperidol (3) Olanzapine (27) Risperidone (4)
**Antidepressants (13)** Amitriptyline (1) Citalopram (3) Mirtazapine (4) Paroxetine (1) Sertraline (4)	**Antidepressants (2)** Mirtazapine (1) Sertraline (1)
**Mood stabilisers (23)** Lamotrigine (1) Lithium (4) Semisodium valproate (1) Sodium valproate (17)	**Mood stabilisers (1)** Lithium (1)
**Sedatives (30)** Clonazepam (19) Diazepam (1) Lorazepam (6) Pregabalin (1) Promethazine (1) Zopiclone (2)	**Sedatives (16)** Clonazepam (15) Lorazepam (1)

## Appendix C

### Ethnic background of sample participants

**Table:**

Ethnicity	Group A (*n* = 140)		Group B (*n* = 88)	
White British	31	22.1%	19	21.6%
Black British Other	20	14.3%	14	15.9%
White other	19	13.6%	12	13.6%
African	12	8.6%	3	3.4%
Other	12	8.6%	5	5.7%
Not stated	11	7.9%	7	8.0%
Black British African	9	6.4%	11	12.5%
Asian British other	5	3.6%	0	0.0%
Black British Caribbean	5	3.6%	3	3.4%
Black other	5	3.6%	7	8.0%
Asian other	3	2.1%	0	0.0%
Asian British Chinese	2	1.4%	2	2.3%
Caribbean	2	1.4%	2	2.3%
Mixed race	2	1.4%	1	1.1%
Pakistani	1	0.7%	0	0.0%
Bangladeshi	0	0.0%	2	2.3%
